# Successful Endoscopic Transsphenoidal Approach Treatment of Sphenoid Sinus Organized Hematoma Causing Visual Deficit: A Case Report

**DOI:** 10.3390/medicina59101802

**Published:** 2023-10-10

**Authors:** Kyu Young Choi, Sun Choi, Suji Jeong, Tae-Bin Won

**Affiliations:** 1 Department of Otorhinolaryngology-Head and Neck Surgery, Kangnam Sacred Heart Hospital, Hallym University College of Medicine, 1 Singil-ro, Yeongdeungpo-gu, Seoul 07441, Republic of Korea; coolq0@hallym.or.kr (K.Y.C.); chl424@naver.com (S.C.); 2 Department of Otorhinolaryngology-Head and Neck Surgery, Seoul National University Hospital, Seoul National University College of Medicine, Seoul 03080, Republic of Korea

**Keywords:** sphenoid sinus, hematoma, visual disorders, transanal endoscopic surgery, nasoseptal flap

## Abstract

*Background*: Organized hematoma in the sphenoid sinus is rare, but serious complications, such as visual deficits, can occur. Three such case reports have been published previously in the literature; however, none have achieved complete recovery of the vision. *Case presentation*: A 69-year-old male patient was referred to the ear, nose, and throat department with an expansile soft tissue mass filling the right sphenoid sinus and blurry vision in his right eye. Complete mass removal was achieved by a wide opening of the sphenoid sinus via an endoscopic transsphenoidal approach, followed by cauterization of the feeding artery and coverage by a nasoseptal flap. The patient’s vision was restored after the operation, and he declared no visual symptoms until the latest follow-up (one year after the surgery). *Conclusions*: Complete excision with an endoscopic endonasal transsphenoidal approach can restore visual deterioration caused by a sphenoid sinus organized hematoma.

## 1. Introduction

A mass filling the sphenoid sinus can be devastating due to the proximity of skull base structures, causing nerve involvement and neurological deficits. Sinonasal organized hematoma is a rare disease that primarily occurs at the maxillary sinus [1,2]; however, five cases of organized hematoma in the sphenoid sinus have been reported to date [1,2,3,4,5]. Although organized hematoma is a slow-growing benign lesion, it demonstrates locally aggressive behavior [2,3,4,5,6]. Among the five cases of sphenoid sinus organized hematoma reported, three presented visual deficits [1,2,3]. While endoscopic surgical resection is reported to be curative for sinonasal organized hematoma, all three cases did not achieve complete recovery of vision after surgical treatment [1,2,3].

Diagnosing organized hematoma is challenging because the symptoms, endoscopic findings, and radiographic findings, mimic other sinonasal diseases and do not provide definite differentiation [5,7]. Even the initial biopsies of these masses are reported to be usually non-diagnostic [5]. Herein, we report a patient with an expansile soft tissue mass filling the right sphenoid sinus with a right visual deficit. After complete removal via an endoscopic transsphenoidal approach and nasoseptal flap, the patient’s vision returned to normal for the first time to be reported in the literature. The pathologic study reported organized hematoma.

## 2. Case Report

**Figure 1:**
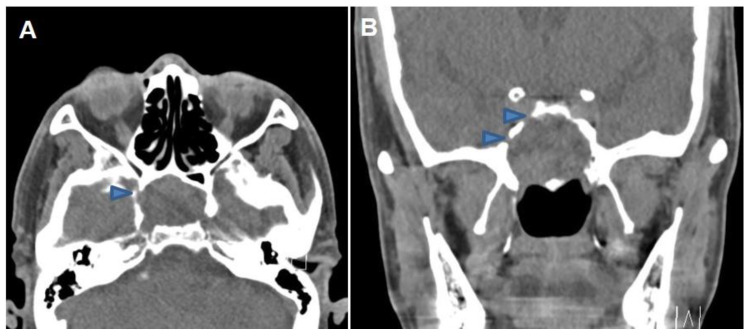
(**A**) Preoperative non-contrast axial and (**B**) coronal CT scans demonstrating space-occupying soft tissue density lesion in the right sphenoid sinus with bony wall erosion (arrowheads).

A 69-year-old male visited the Ophthalmology clinic with two transient episodes (duration less than one minute) of right-side visual disturbance (blurry vision). The ophthalmologist declared no specific lesion in both eyes, so he was transferred to the Neurosurgery department. Magnetic resonance imaging and angiography (MRI and MRA) of the brain were taken that showed tight stenosis at the right proximal internal carotid artery (ICA) and a heterogeneous mass lesion in the sphenoid sinus. Common carotid angiography was performed that revealed 70% of stenosis at the right proximal ICA, so he was prescribed Cilostan^®^. Then, he was transferred to the Otolaryngology department to evaluate the sphenoidal mass. Paranasal sinus computed tomography (CT) revealed a soft tissue density lesion occupying the right sphenoid sinus (3.3 × 2.7 × 2.2 cm^3^) with a sclerotic wall and mid-cranial fossa defect (Figure 1). An endoscopic biopsy was planned; after sphenoidotomy using back-biting rongeur, the sphenoidal mass was ripped off and sent for pathologic study. A significant volume of hemorrhagic content was drained out, and the patient declared no specific symptoms for his eye after the surgery. The surgical biopsy was reported to be non-diagnostic (inflammatory with increased elastic fiber and dystrophic calcification).

**Figure 2:**
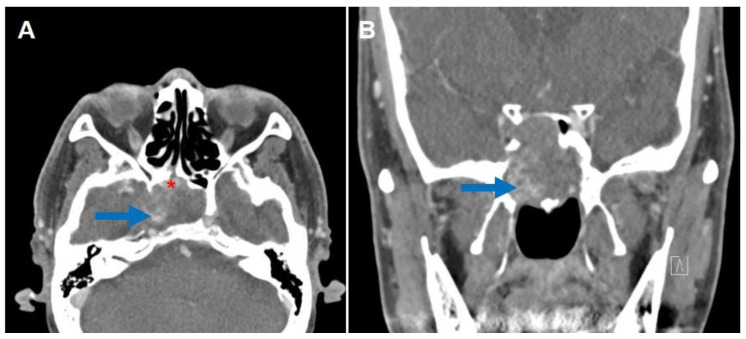
(**A**) Contrast-enhanced axial and (**B**) coronal CT scans show an enlarged expansile mass in the right sphenoid sinus with focal patchy enhancement (arrows). A previous sphenoidotomy site is also seen (*).

**Figure 3:**
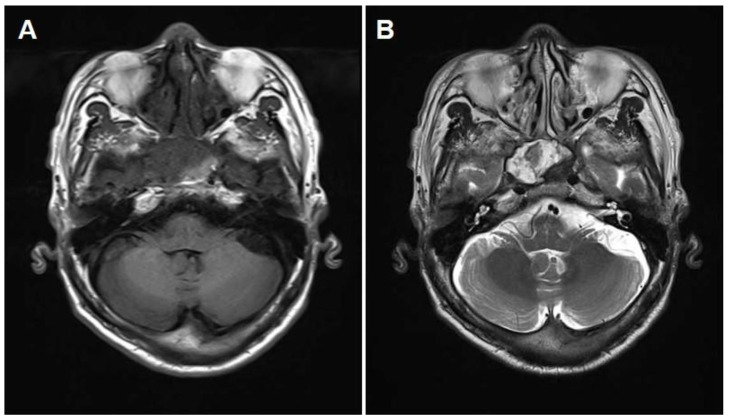
(**A**) T1-weighted MRI demonstrating an expansile mass filling the right sphenoid sinus with heterogeneously mixed signals. (**B**) T2-weighted MRI showing marked heterogeneous signal intensity and hypointense peripheral rim.

The patient was well until one year and three months after the surgical biopsy and drainage when he declared relapsed blurry vision in his right eye that continued after three transient episodes of right-side visual disturbance. Eye examination revealed intact extraocular movement on both sides; however, visual acuity of the right eye was decreased to 0.15 compared to the left with 1.0. Follow-up CT (Figure 2) and MRI (Figure 3) were taken, which revealed a right sphenoid sinus with expansile mass with patchy enhancement and heterogeneous signal intensity. The patient did not declare any other symptoms such as headache, facial pain, nasal obstruction, epistaxis, etc.

**Figure 4:**
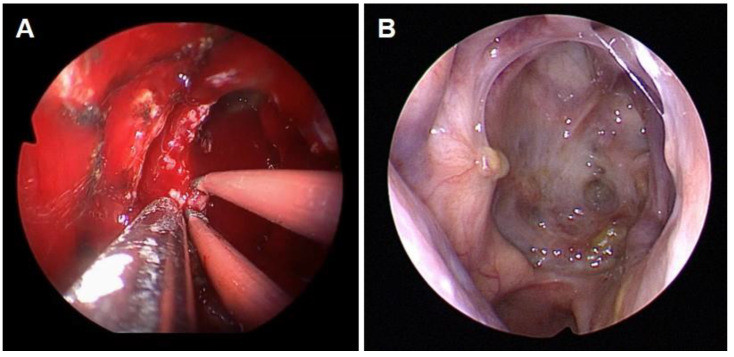
(**A**) Intraoperative endoscopic view of the right sphenoid sinus and cauterization of the feeding artery of the organized hematoma. (**B**) Postoperative endoscopic view.

**Figure 5:**
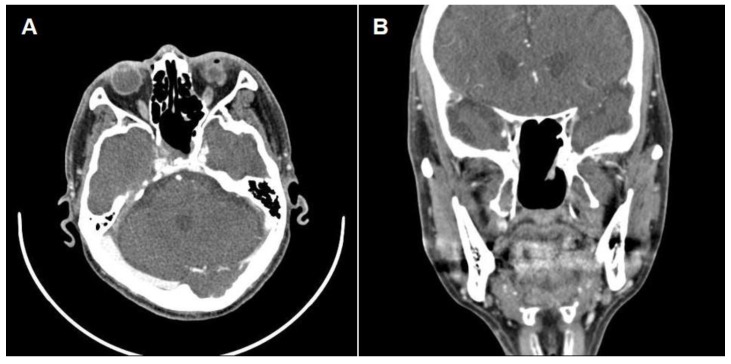
(**A**) Postoperative axial and (**B**) coronal CT scans reveal complete excision of organized hematoma at the right sphenoid sinus.

The patient was referred to a tertiary hospital with a dedicated skull base center for the complete removal of the sphenoidal mass and decompression of the optic nerve. The surgery was performed via a binostril approach identical to an endoscopic endonasal transsphenoidal approach under image guidance. The septal mucosal was elevated on the left side via a hemitransfixion incision. A rescue incision was made on the right septum enabling binostril surgery. The posterior bony septum and sphenoid rostrum were removed allowing wide access to both sphenoid sinuses. The mass was fragile with dark purple coloration. Complete resection of the mass was performed in a piece-by-piece manner using forceps and curettes. Significant and continuous bleeding was encountered during mass debulking, gradually subsiding during resection. A fibrotic capsule was identified abutting the skull base and lateral wall of the sphenoid sinus. The bone was mostly eroded, and the capsule was carefully peeled off, leaving the dura intact. After complete removal, a bleeding vessel was identified in the floor of the right sphenoid sinus, which was determined to be the vidian artery, that was cauterized with a bipolar and suction bovie (Figure 4A). A vascularized nasoseptal flap was raised from the right septum at the end of the surgery to cover the floor and exposed lateral wall of the sphenoid sinus. Histopathology examination reported hemorrhagic regions surrounded by fibrous tissues and neovascularization, consistent with an organized hematoma. The patient declared restored vision right after the surgery. Postoperative endoscopy and CT revealed complete resection of the hematoma (Figure 4B and Figure 5). The patient’s vision was still normal at the latest follow-up (one year after the surgery).

## 3. Discussion

Organized hematoma is known to result from an initial hemorrhagic event caused by various etiologies that progress with the organization of the contents through fibrosis and neovascularization [1,4]. Without reabsorption within a poorly ventilated sinus, the hematoma expands with recurrent intracapsular bleeding [1,4]. Predisposing factors such as bleeding diathesis, antiplatelet medications, and anticoagulation can lead to intracapsular bleeding with organization of the accumulated blood [1,3,8,9]. In our case, the patient also took antiplatelet medicine. Giant et al. have reported a case of organized hematoma that revealed growth and expansion of the mass after rebleeding from the initial surgery [1]. They reported that failure to identify the source of bleeding might lead to further growth and recurrence. In this case, the source of bleeding was not identified in the first surgery; however, a wide opening of the sphenoid sinus utilizing the transsphenoidal approach led to the identification and cauterization of the feeding vessel that suppressed the recurrence of the organized hematoma and related symptoms.

With an increasing incidence of paranasal sinus organized hematomas [10], the symptoms include epistaxis, nasal obstruction, headache, facial pain, and, less commonly, other compressive symptoms [1,11]. Most sinonasal organized hematomas occur within the maxillary sinus; however, organized hematomas in the sphenoid sinus can lead to more severe complications than those in the maxillary sinus due to the relatively small volume of the sinus and the anatomical proximity to the skull base [1,2,3]. In this case, the organized hematoma almost completely filling the right sphenoid sinus is thought to cause a compressive effect on the optic nerve in the sphenoid sinus that yielded the visual deficit. Although it is classified as a benign disease, the expansive and destructive nature of an organized hematoma necessitates differential diagnosis with other aggressive diseases such as cancer, inverted papilloma, mucocele, etc. [3].

While radiologic studies are helpful, histological evaluation is necessary for the definite diagnosis of an organized hematoma [12]. Mixtures of fibrosis, neovascularization, hemorrhage, and extravasated red blood cells are the histopathological findings of organized hematomas [2,12,13]. Correct pre-operative diagnosis is challenging because the symptoms and imaging findings are relatively nonspecific [2,4,14,15]. They are often mistaken as malignancies due to their aggressive clinical appearance and diagnostic imaging findings that favor advanced disease [16,17]. CT findings of sinonasal organized hematomas include expansile mass with heterogenous patchy enhancement in post-contrast images causing adjacent bony changes [2,5,18,19]. MRI findings include a well-demarcated mass from the surrounding structures with nodular and patchy enhancement on post-contrast T1-weighted imaging and marked heterogeneous signal intensity in T2-weighted imaging with the hypointense peripheral rim indicating the fibrous capsule [3,5,20,21].

In this case, inverted papilloma could be ruled out by the absence of hyperostotic change often seen at the attachment point on CT and by the lack of a classic cerebriform pattern of inverted papilloma on the MRI [5]. The differential diagnosis of malignant neoplasm can be made by the absence of clinical findings such as pain or bleeding and by the lack of apparent invasion of adjacent tissue on imaging studies. Mucocele, inflammatory polyp, cholesterol granuloma, fungus ball, and hemangioma can all be differentiated from an organized hematoma by the different enhancement patterns on a CT [18].

An organized hematoma arising in the sphenoid sinus was first reported by Nakagawa et al. in 2010 [4]. Since then, four more cases of sphenoid sinus organized hematoma have been reported in the literature [1,2,3,5]. Sphenoidal sinus organized hematoma that arouses visual loss was first reported by Yoon et al. in 2018 [3]. Although they performed emergent endoscopic surgery, the patient’s visual symptoms did not improve. The differential diagnosis of organized hematoma in patients for sphenoid sinus lesions with acute visual loss and timely treatment has been emphasized [3]. A sphenoidal organized hematoma with acute vision loss has been successfully treated by Lin et al.; by an expanded endonasal transpterygoid approach and debulking, the vision was recovered partially but not wholly [1]. A sphenoidal organized hematoma causing multiple cranial neuropathies (optic and oculomotor neuropathy) has also been reported [2]. After endoscopic endonasal surgery, oculomotor neuropathy and severe headache were recovered; however, visual loss remained. These cases have been well summarized for the symptoms, imaging features, and outcomes in a table that Lin et al. reported [1].

Although successful treatment relies on complete surgical excision [2,4,22,23], complete resection of an organized hematoma in the sphenoid sinus is relatively tricky due to the proximity of vital organs and massive intraoperative bleeding. This case was the first to be reported in the literature that completely recovered visual defect after surgical treatment of a sphenoidal organized hematoma. Complete excision of the mass utilizing the transsphenoidal approach and nasoseptal flap restored the patient’s vision without sequela. Although the recommended time for the decompression of compressive optic neuropathy is reported as 24 h, the patient, in this case, completely recovered his vision after two months of the onset of the symptom via surgery.

The endoscopic endonasal transsphenoidal approach has been widely used recently for the resection of skull base lesions [24], and the use of pedicled nasoseptal flap for the reconstruction of skull base defect has been reported as the most valuable method that reduces the incidence of CSF leakage to less than 5% [25]. However, much experience is needed to perform the procedure without any neurovascular damage and to achieve a watertight closure of the skull base defect by harvesting a sufficiently sized pedicled nasoseptal flap. This case highlights the need for a wide opening of the sphenoid sinus via an endoscopic transsphenoidal approach for complete resection of the sphenoid sinus organized hematoma causing compressive neuropathy. Complete mass removal, followed by cauterization of the feeding artery and coverage by the nasoseptal flap, fully restored the visual deterioration of the patient without recurrence, for the first time to be reported.

As organized hematomas are often misdiagnosed preoperatively, precise CT and MRI diagnosis is recommended to avoid any perioperative complication or excessive surgical intervention. The need for experience in endoscopic skull base surgery for rhinologists to implement appropriate and timely intervention of sphenoid sinus organized hematomas causing compressive neuropathy is also emphasized. The limitation of this case report includes the absence of histopathologic images, which have been extensively reported previously [1,3,4,5,6,7]. Failure to completely remove the organized hematoma at the first surgery can also be criticized.

## 4. Conclusions

Organizing hematomas should be suspected in the case of visual deterioration with a mass filling the sphenoid sinus. Complete resection via the endoscopic endonasal transsphenoidal approach and cauterization of the feeding artery, followed by a nasoseptal flap, is recommended to treat an organizing hematoma of the sphenoid sinus and related neurological symptoms.

## Data Availability

The original data presented in this study are available on request from the corresponding author.
